# Mechanical Properties and Epoxy Resin Infiltration Behavior of Carbon-Nanotube-Fiber-Based Single-Fiber Composites

**DOI:** 10.3390/ma14010106

**Published:** 2020-12-29

**Authors:** Jongseon Shin, Kyunbae Lee, Yeonsu Jung, Byeongjin Park, Seung Jae Yang, Taehoon Kim, Sang Bok Lee

**Affiliations:** 1 Department of Chemistry and Chemical Engineering, Education and Research Center for Smart Energy and Materials, Inha University, Incheon 22212, Korea; 22181525@inha.edu (J.S.); sjyang@inha.ac.kr (S.J.Y.); 2 Composites Research Division, Korea Institute of Materials Science, Changwon 51508, Korea; kblee@kims.re.kr (K.L.); ysjung@kims.re.kr (Y.J.); b.park@kims.re.kr (B.P.)

**Keywords:** CNT fiber, composite, epoxy, infiltration, mechanical properties

## Abstract

Carbon nanotube fiber (CNTF), prepared by the direct-spinning method, has several nanopores, and the infiltration behavior of resins into these nanopores could influence the mechanical properties of CNTF-based composites. In this work, we investigated the infiltration behavior of resin into the nanopores of the CNTFs and mechanical properties of the CNTF-based single-fiber composites using six epoxy resins with varying viscosities. Epoxy resins can be easily infiltrated into the nanopores of the CNTF; however, pores appear when a resin with significantly high or low viscosity is used in the preparation process of the composites. All the composite fibers exhibit lower load-at-break value compared to as-densified CNTF, which is an unexpected phenomenon. It is speculated that the bundle structure of the CNTF can undergo changes due to the high affinity between the epoxy and CNTF. As composite fibers containing pores exhibit an even lower load-at-break value, the removal of pores by the defoaming process is essential to enhance the mechanical properties of the composite fibers.

## 1. Introduction

Carbon nanotube fibers (CNTFs) show high electrical conductivity, high thermal conductivity, high strength, and are lightweight because they are composed of only carbon nanotubes (CNTs) [1,2]. The CNTFs can be fabricated by the following three methods: forest spinning [3], solution spinning [4], and direct-spinning [5]. Among these three fabrication methods, direct-spinning is the most suitable method to prepare continuous CNTFs possessing several nanopores. Further, functional CNTFs can be developed by the incorporation of functional materials into the nanopores of the CNTF. For example, fiber-type supercapacitor [6], lithium-ion battery [7], and thermoelectric generators [8], based on direct-spun CNTFs, have been reported. Moreover, the strength of the direct-spun CNTF can be enhanced by post treatments [5,9], leading to super-strong fibers that have higher specific strength than the carbon fiber (CF) T1000, which is known as the strongest commercial carbon fiber [5]. Therefore, direct-spun next-generation fibers simultaneously exhibit strength, functionality, and productivity.

CF is mostly fabricated as fiber-reinforced plastic (FRP) for use in structural materials. Likewise, CNTF should also be fabricated in the form of FRP for use as a light and strong structural material. There have been a few studies on FRP, based on direct-spun CNTF; however, studies on FRP using CNTF and CNT sheets prepared by the three above-mentioned methods have been reported by several groups [10,11]. Various matrix resins, including epoxy [12,13], bismaleimides (BMI) [14], polyvinyl alcohol (PVA), ref. [15] have been used to prepare the composites. PVA-based composites generally exhibit high strength, whereas the epoxy-based composites showed relatively low mechanical properties [16]. We reported that the composite fibers, prepared by the infiltration of PVA, exhibited higher specific strength than the infiltration of polyacrylonitrile (PAN) and polystyrene (PS) [17]. While many studies support that PVA is a suitable material for matrix to obtain composites with high strength, the most widely used matrix material in the CF-reinforced plastic (CFRP) is epoxy. This is because it has high stiffness, excellent thermal and chemical stabilities, and is relatively easy to process through the curing process. Therefore, epoxy will be used in the preparation of CNTF-reinforced plastic (CNTFRP) in the future. However, the relatively low strength of epoxy-based CNTFRP should be improved.

The tensile strength of the FRP is generally determined by the tensile strength of the fibers, rather than that of the matrix. However, in the case of CNTF, the infiltration of the polymer matrix into the nanopores of the CNTF is known to change the internal structure and mechanical properties of the CNTF [17]. We assume that the infiltration of the epoxy will also impair the mechanical properties of the CNTF. Moreover, epoxy has a higher viscosity than dilute polymer solutions used in the previous studies [17], preventing the infiltration of the epoxy into the nanopores of the CNTF and influencing the mechanical properties of the CNTF. However, few studies have been performed to examine the nanostructure of CNTFRP, based on the epoxy matrix. In particular, many studies on the CNTFRP have been performed using forest-spun CNTF rather than direct-spun despite their different nanostructures.

In this work, we investigated the infiltration behavior of epoxy resin into the nanopores of direct-spun CNTF and its effect on the mechanical properties. To study the infiltration feature of an epoxy resin into the CNTF, we altered the viscosities of the epoxy resins, analyzed the cross-section images of the composite fibers, and measured the tensile strength. The epoxy resin was effectively infiltrated into the nanopores without any defect when epoxy resins with appropriate viscosities were used.

## 2. Materials and Methods

### 2.1. Materials

Ferrocene, thiophene, N,N′-dimethyl benzyl amine (BDMA), and methyl tetrahydrophthalic anhydride (MeTHPA) were purchased from Sigma Aldrich (St. Louis, MO, USA). Acetone was purchased from Samchun Chemical (Seoul, Korea). Bisphenol A diglycidyl ether (DGEBA) and diethyltoluenediamine (DETDA) were provided by Kukdo Chemical (Seoul, Korea). All the chemicals were used without further purification.

### 2.2. Synthesis of Direct-Spun CNTF

Direct-spun CNTFs were synthesized at 1200 °C using the floating catalyst chemical vapor deposition method, as described in our previous study [5]. In this study, a continuously produced CNT aerogel was transformed into a fiber by passing it through a water bath. The as-synthesized CNTF was densified by dipping it in acetone.

### 2.3. Preparation of Composite Fibers

**Figure 1:**
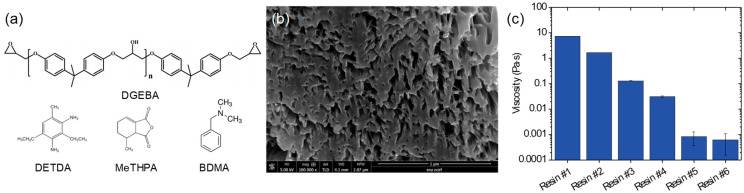
(**a**) Chemical structures of the epoxy resin and curing agents. (**b**) Cross-section image of as-densified CNTF. (**c**) Viscosities of the six resins used in this work.

To study the infiltration behavior of the epoxy resins into the nanopores of the CNTF, we prepared six epoxy resin systems (Resins #1–6; see Table 1) with varying viscosities. The resins were composed of DGEBA, hardener, and with or without acetone. DGEBA was chosen as the common epoxy resin for the six epoxy resin systems because of its good mechanical properties. Two curing agents, i.e., DETDA and MeTHPA, were used to prepare Resins #1 and #2, respectively, to control the viscosity of the epoxy. The hardeners had a lower viscosity than the epoxy (DGEBA). An equivalent ratio of DGEBA: DETDA is 4:1 and that of DGEBA:MeTHPA is 10:9. Therefore, the combination of DGEBA and MeTHPA has lower viscosity. Controlling the viscosity of an epoxy using curing agents has previously been reported [18]. The chemical agents used in this work are shown in Figure 1a.

The CNTFs were dipped in the mixed epoxy resins for 1 min to impregnate the resin, and the excess resin was removed by squeezing the fibers. The defoaming process was performed by storing the resin-impregnated fibers in vacuum at 25 °C for 30 min to remove the micro air bubbles in the fibers. The composite fibers were named as CNTFRP-n when the CNTFs were dipped in Resin #n. CNTFRP-1 was cured at 120 °C for 2 h, 150 °C for 1 h, and 180 °C for 2 h. The other CNTFRPs were cured at 80 °C for 2 h, 120 °C for 1 h, and 150 °C for 2 h.

### 2.4. Characterization

The internal structures of the CNTFRPs were observed after cutting the material with a focused ion beam (Helios 650, FEI, Hillsboro, OR, USA). The shear viscosity of the resin was measured with a rheometer (Anton Paar physica MCR 302, Graz, Austria) equipped with a parallel plate geometry (diameter 25 mm and gap 1 mm) at shear rates of 20 s^−1^. The mechanical properties were measured by Dynamic Mechanical Analyzer (TA Instrument Q800, New Castle, DE, USA) at a force rate of 0.05 N/min. The gauge length of the test sample was 1 cm. The strengths of the fibers and composite fibers were not reliable because the cross-section area was not accurate; therefore, the specific strength was used in this study for both the CNTF and composite fibers. The linear density was measured by measuring the weight of 10 m of the fibers.

## 3. Results and Discussion

The diameter and linear density of the direct-spun CNTF synthesized in this work were approximately 13 ± 1.1 µm and 0.067 ± 0.005 tex, respectively. The cross-section image of the CNTF showed that the CNTF had numerous nanopores of the size of tens of nanometers (Figure 1b). Six sets of epoxy resins were applied to observe the effects of the resins’ viscosity. The combination of DGEBA and DETDA (Resin #1) exhibited higher viscosity (~7 Pa·s, see Figure 1c) than the combination of DGEBA and MeTHPA (Resin #2, ~1.3 Pa·s). Resins #3–#6 were prepared by adding acetone to Resin #2 to decrease the viscosity of the resin. Resins #3 and #4 showed viscosities of 0.13 Pa·s and 0.03 Pa·s, which are one and two orders smaller than that of Resin #2, respectively. The viscosities of Resins #5 and #6 were similar to that of organic solvents (~0.001 Pa·s), which is similar with the previous studies on the infiltration of dilute polymer solutions [17]. To sum up, Resins #1–#4 can reveal the effect of viscosity and the addition of a solvent on the infiltration and mechanical properties of the composite fibers. Resins #5 and #6 can show the change in the mechanical properties after the infiltration of the dilute epoxy solution and curing.

**Figure 2:**
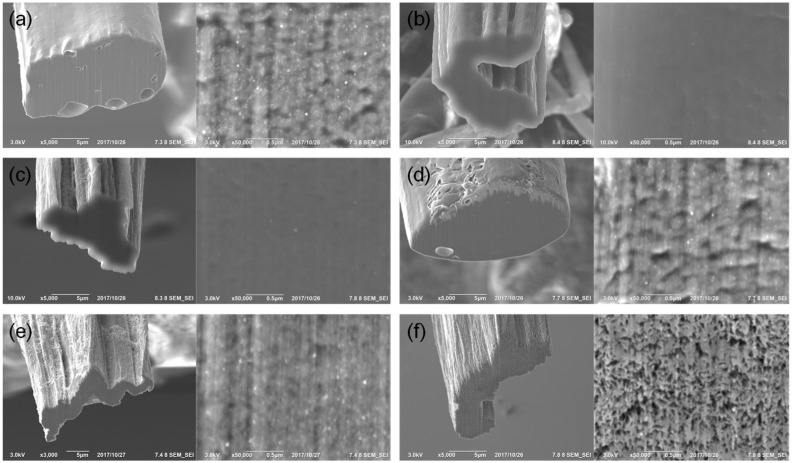
Cross-section images of CNTFs with infiltrated (**a**) Resin #1, (**b**) Resin #2, (**c**) Resin #3, (**d**) Resin #4, (**e**) Resin #5, and (**f**) Resin #6. The left images are low-magnification images and the right images are high-magnification images.

We infiltrated the epoxy resins into the CNTF, and subsequently cured and observed the cross-sections of the composite fibers (Figure 2). Contrary to the prediction that the resin will be difficult to infiltrate into the nanopores of the CNTF, it can be observed that Resin #1, which has the highest viscosity, was well infiltrated into the nanopores, while a few macropores were created. In the case of the dilute epoxy resin, the nanopores remained after infiltration and curing due to the high ratio of the volatile solvent (Figure 2e,f). Infiltration without both the nanopores and macropores was achieved when the epoxy resins with viscosities of 0.1–2 Pa∙s were infiltrated. When the viscosity of the resin was below 0.1 Pa∙s, the nanopores were not filled with the resins. In particular, when Resin #6 was used, the nanostructure of the CNTF was similar to the as-densified CNTF, except for the resin-rich region that was observed. Therefore, the viscosities of the resins for the preparation of CNTFRP should be 0.1–2 Pa∙s, and the addition of a small amount of solvent is desirable, while the addition of a large amount (>50 wt%) of solvent prevented the filling of the pores with the resin.

**Figure 3:**
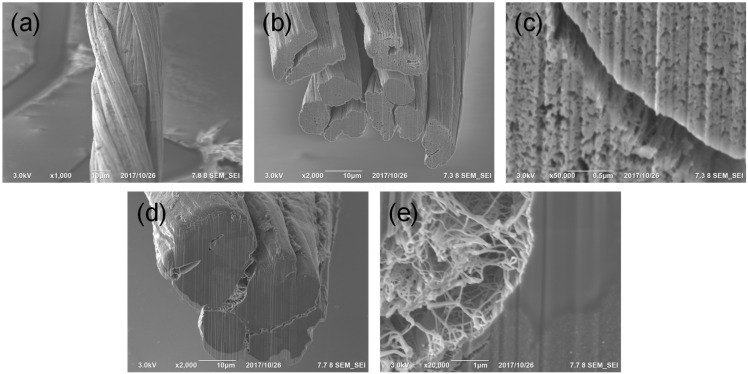
(**a**) Scanning electron microscope image, (**b**) cross-section image, and (**c**) high-magnification image of the as-twisted multifilament CNTF. (**d**) Cross-section image and (**e**) high-magnification image of Resin #2-infiltrated multifilament CNTF.

To confirm the large-scale infiltration of the resin into the nanopores of the CNTFs, we prepared multifilament CNTFs by twisting 10 single filaments (see Figure 3a). Figure 3b,c are the cross-sectional images of the multifilament CNTFs, showing the same nanopores structure of the CNTFs and change in the fiber shape due to the pressure between the filaments. The change in the fiber shape will be beneficial for the preparation of high-performance composite materials in future because it can increase the volume fraction of a fiber in a composite. Since Resin #2 was effectively infiltrated into the nanopores of the single-filament CNTF, we impregnated the multifilament CNTFs in Resin #2 and cured the composite fibers. Figure 3d,e show the cross-sectional images of the composite fibers, exhibiting the excellent infiltration of the resin into the nanopores of the multifilament fibers. The cracks in the Figure 3d were not induced by poor infiltration behavior of the resin, but induced by poor contact between the single-filament CNTFs since the curing process was performed on a freestanding state without pressing. In the future, when the CNTF fabric will be used in the fabrication of composites, the gap will be simply removed because a high pressure will be applied during a common composite fabrication process such as press molding, vacuum assisted resin transfer molding, and autoclave process. This result indicates that the resin is infiltrated not only into the nanopores of the single filament with a diameter of 10 um, but also into the nanopores of the multifilament and even the CNTF fabric. Therefore, we can conclude that the resin infiltration will not be an issue in the preparation of CNTFRP.

**Figure 4:**
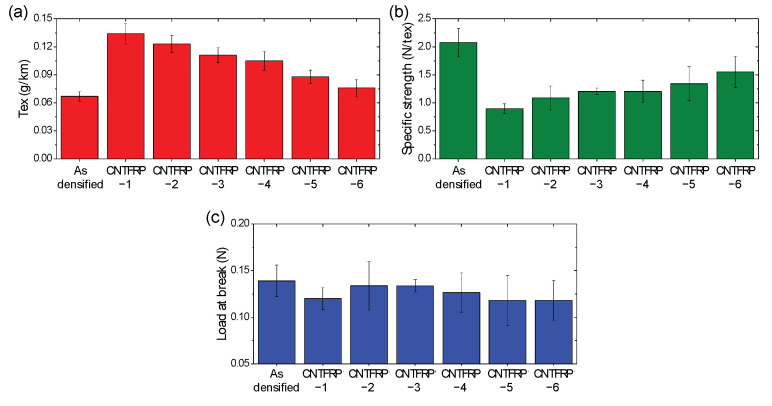
(**a**) Linear density, (**b**) specific strength, and (**c**) load-at-break value of the as-densified CNTF and composite fibers.

To analyze the mechanical properties of the CNTF before and after composite processing, linear density, specific strength, and load-at-break values of the composites fibers were measured (Figure 4). As expected form the cross-section images (Figure 2), the linear density of the composite fibers increased when a highly viscous resin was used. It seems that the resin with a low viscosity is easier to be squeezed out. Further, the linear density of the CNTFRP-6 was similar to that of the as-densified CNTF since only a small amount of resin remained after 99% of acetone was evaporated. The trend of specific strength of the composite fibers was opposite to that of the linear density. The specific strength of the resin (80 MPa, 1.23 g/cm^3^, 0.065 N/tex) is significantly smaller than that of the CNTF (2.1 N/tex); hence, the more resin remaining in the composite fibers, the lower the specific strength is observed. Figure 4c shows the applied load value at the break point of the fibers, meaning the point at which the fibers can withstand the tension. The addition of the resin to the composite fibers should increase the load-at-break value because both CNTF and epoxy resin can withstand the tension. However, Figure 4c show that the load-at-break value decreased after infiltration of the resins regardless of the type of the resins. CNTFRP-1 showed a large reduction in load-at-break value (0.139 N to 0.120 N). In contrast, CNTFRP-2 and CNTFRP-3, which showed a flawless internal structure, exhibited higher load-at-break value (both 0.134 N) than CNTFRP-1. CNTFRP-4 possess some nanopores after resin infiltration, which might influence the load-at-break value (0.127 N). It seems that the pores (~50 nm) inside the composite fibers decreases the load-at-break value. The composite fibers prepared by the infiltration of dilute resin solutions, i.e., CNTFRP-5 and CNTFRP-6, exhibited high specific strength (1.34 N/tex and 1.55 N/tex, respectively) since the increase in the linear density of the fibers after resin infiltration was not high. However, the load-at-break value of the composite fibers are significantly lower than that of the as-densified CNTF (both 0.118 N). Contrary to the previous results that showed enhanced strength compared to as-prepared CNTF after infiltration of dilute polymer solutions into CNTF [17], infiltration of a dilute epoxy solution induced negative effects on the mechanical properties of the CNTF. Our previous results demonstrated that PVA with a low affinity with CNT increased the strength of the CNTF by increasing the diameter of the bundles [17]. In contrast, the PS that has a high affinity with CNT decreased the strength because the polymer with a high affinity with CNT can change the bundle structure of the CNTF, which worsens the mechanical properties. In this study, we speculated that the epoxy and CNTs have a good affinity because the epoxy resin was well impregnated into the nanopores of the CNTF without any post-treatment. Furthermore, we presume that such a high affinity between the epoxy resin and CNTs lowered the strength of CNTFRP-5 and CNTFRP-6 as the PS also lowered the strength of the CNTF. The internal bundle structure of CNTFRP-1, -2, -3, and -4 were also changed by the infiltration of the epoxy, which might be the reason for the decrease in the load-at-break value of the CNTFRP.

**Figure 5:**
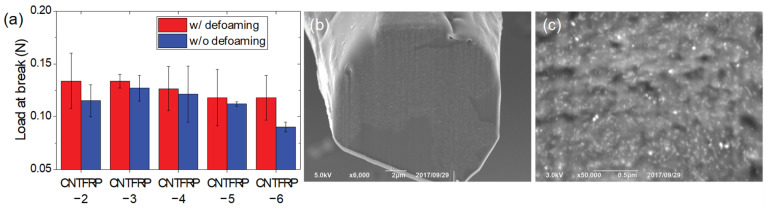
(**a**) Load-at-break value of the composite fibers prepared with defoaming (red) and without defoaming (blue) process. (**b**) Cross-section and (**c**) high-magnification images of CNTFRP-2 without defoaming.

In this study, all the composite fibers that are discussed above were prepared with the defoaming process. We also prepared the composite fibers without the defoaming process to observe the effect of the defoaming process. Figure 5 shows the load-at-break value of the composites fiber with and without the defoaming process. Regardless of the type of resin, the load-at-break values were reduced. The reason for this observation can be found in the cross-section image (see Figure 5b,c). Figure 5b,c show the internal structure of CNTFRP-2 prepared without the defoaming process. Contrary to CNTFRP-2 prepared with the defoaming process (see Figure 2b), the internal nanopores were observed when the defoaming process was not applied. Since the internal pores reduce the load-at-break value of the composite fibers, the defoaming process is necessary during the fabrication of the CNTFRP.

## 4. Conclusions

We investigated the infiltration behavior of epoxy resin into a direct-spun CNTF, and studied the mechanical properties of the CNTF-based single-fiber composites. Without post-treatment, the epoxy resin can easily impregnate the nanopores of the CNTF, and the optimum viscosity of the resin was 0.1–2 Pa·s. When the resin with high viscosity was used, it is well infiltrated into the nanopores, but generates macropores. In contrast, when the acetone-added resins having low viscosity were used, the nanopores of the composite fibers were observed owing to the evaporation of acetone. All the composite fibers prepared in this work showed a reduced specific strength compared to the as-densified CNTF, which is due to the increased linear density of the CNTF. Interestingly, the load-at-break values of the single fibers also decreased after composite fabrication. We speculated that the high affinity between the epoxy resin and the CNTF changed the internal bundle structure of the CNTF, thereby resulting in the reduced tensile strength of the single-fiber composite. Since both macropores and micropores decreased the load-at-break value of the CNTFRP, the suitable resin for obtaining high load-at-break value is one that can fill nanopores without generating macropores. This suggests that the viscosity of the resin should be 0.1–2 Pa·s. Moreover, defoaming is essential for obtaining a high load at break since the process removes the pores in the composite fibers. This work will be helpful in the development of direct-spun CNTF-based composites, and provide the physical insights on the high-performance CNTFRP.

## Figures and Tables

**Table 1:** Summary of the epoxy resin systems used in this work.

Code	Resin	Curing Agent	Solvent	Ratio of Resin (wt.%)	Ratio of Curing Agent (wt.%)	Ratio of Solvent (wt.%)	Viscosity (Pa·s)
Resin #1	YD128	DETDA	-	80	20	-	7.28
Resin #2	YD128	MeTHPA + BDMA	-	52.2	47.8	-	1.66
Resin #3	YD128	MeTHPA + BDMA	Acetone	43.5	39.8	16.7	0.13
Resin #4	YD128	MeTHPA + BDMA	Acetone	26.1	23.9	50	0.03
Resin #5	YD128	MeTHPA + BDMA	Acetone	5.2	4.8	90	<0.001
Resin #6	YD128	MeTHPA + BDMA	Acetone	0.52	0.48	99	<0.001

## Data Availability

The data presented in this study are available on request from the corresponding author.
